# *S*‐adenosyl‐L‐homocysteine extends lifespan through methionine restriction effects

**DOI:** 10.1111/acel.13604

**Published:** 2022-04-07

**Authors:** Takafumi Ogawa, Koji Masumura, Yuki Kohara, Muneyoshi Kanai, Tomoyoshi Soga, Yoshikazu Ohya, T. Keith Blackwell, Masaki Mizunuma

**Affiliations:** ^1^ 12803 Unit of Biotechnology Graduate School of Integrated Sciences for Life Hiroshima University Higashi‐Hiroshima Japan; ^2^ 12803 Hiroshima Research Center for Healthy Aging (HiHA) Hiroshima University Higashi‐Hiroshima Japan; ^3^ Joslin Diabetes Center Harvard Stem Cell Institute, and Harvard Medical School Department of Genetics Boston Massachusetts USA; ^4^ National Research Institute of Brewing Higashi‐Hiroshima Japan; ^5^ Institute for Advanced Biosciences Keio University Tsuruoka Japan; ^6^ Department of Integrated Biosciences Graduate School of Frontier Sciences The University of Tokyo Kashiwa Japan

**Keywords:** *Caenorhabditis elegans*, methionine restriction (MetR), *Saccharomyces cerevisiae*, *S*‐adenosyl‐L‐homocysteine (SAH), *S*‐adenosyl‐L‐methionine (SAM)

## Abstract

Methionine restriction (MetR) can extend lifespan and delay the onset of aging‐associated pathologies in most model organisms. Previously, we showed that supplementation with the metabolite *S*‐adenosyl‐L‐homocysteine (SAH) extends lifespan and activates the energy sensor AMP‐activated protein kinase (AMPK) in the budding yeast *Saccharomyces cerevisiae*. However, the mechanism involved and whether SAH can extend metazoan lifespan have remained unknown. Here, we show that SAH supplementation reduces Met levels and recapitulates many physiological and molecular effects of MetR. In yeast, SAH supplementation leads to inhibition of the target of rapamycin complex 1 (TORC1) and activation of autophagy. Furthermore, in *Caenorhabditis elegans* SAH treatment extends lifespan by activating AMPK and providing benefits of MetR. Therefore, we propose that SAH can be used as an intervention to lower intracellular Met and confer benefits of MetR.

## Graphical abstract

A model depicting how SAH extends lifespan. The intake of SAH reduces intracellular Met and induces benefits associated with MetR in yeast and nematodes. SAH treatment extended lifespan via inhibition of mTORC1 and activation of autophagy and AMPK.

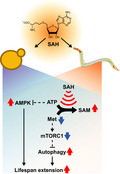

1

Dietary restriction, including MetR, is an effective strategy for promoting longevity and counteracting age‐related morbidities (Ables & Johnson, 2017; Parkhitko et al., 2019). In addition, genetic manipulation or pharmacological inhibition of Met metabolic pathways (Annibal et al., 2021; Hepowit et al., 2021; Johnson & Johnson, 2014; Obata & Miura, 2015; Ogawa et al., 2016; Ruckenstuhl et al., 2014) and a Met‐restricted diet prolong lifespan (Orentreich et al., 1993; Wu et al., 2013). Several studies indicate that a MetR diet is possible for humans (Dong et al., 2018; Gao et al., 2019; McCarty et al., 2009; Olsen et al., 2020, 2021), but long‐term compliance to such a diet is considered problematic. Previously, we showed that a yeast mutant that accumulates *S*‐adenosyl‐L‐methionine (SAM) to high levels exhibited reduced intracellular Met and lifespan extension mediated through AMPK activation (Ogawa et al., 2016) (Figure 1a). We also showed that in a wild‐type (WT) strain, supplementation with SAH increased SAM levels, activating AMPK, and extending lifespan. However, we did not determine whether SAH supplementation might be sufficient to reduce Met levels or determine how SAH supplementation leads to SAM accumulation and lifespan extension.

**FIGURE 1:**
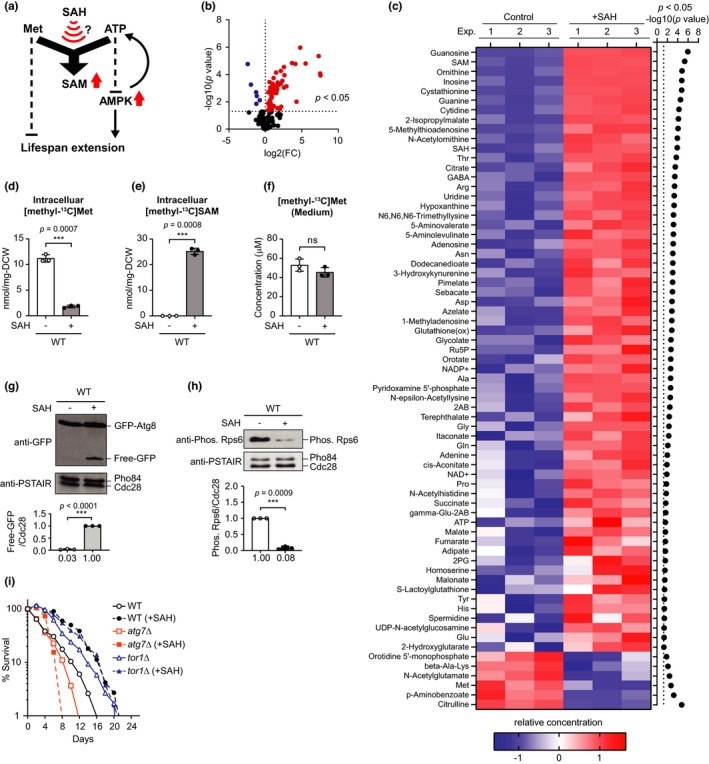
SAH reduces intracellular Met and induces MetR‐like conditions in *S. cerevisiae*. (a) Model for yeast longevity mediated by the stimulation of SAM synthesis by SAH. Volcano plot (b) or heat map (c) showing metabolite levels in WT cells with or without SAH supplementation. *n* = 3. FDR < 0.05, two‐sided unpaired *t*‐test. See also in Table S1. Intracellular [methyl‐^13^C]Met (d), Intracellular [methyl‐^13^C]SAM levels (e), and [methyl‐^13^C]Met levels in the medium (f) were assessed using CE‐TOFMS. Mean ± S.D, *n* = 3, two‐sided unpaired *t*‐test. The relative intensity of free GFP (g) or phosphorylated Rps6 (h) normalized to Cdc28 is shown. Mean ± SD, *n* = 3, two‐sided unpaired *t*‐test. (i) The CLS curve is indicated. (d–f, g, h) ns, not significant; ***p < 0.001. (i) Statistical analyses are shown in Table S2

To investigate the basis for SAH‐mediated longevity, we performed metabolomics (CE‐TOFMS) analysis of a WT *S*. *cerevisiae* strain. In response to 1 mM SAH, which can extend lifespan (Ogawa et al., 2016), 148 metabolites were detected (Table S1). Of these metabolites, 63 and 6 were significantly up‐regulated and down‐regulated, respectively (Figure 1b,c). As previously reported, SAH administration increased levels of SAH and SAM, a methyl group donor (Ogawa et al., 2016) (Figure 1c and Figure S1a). SAH is a potent competitive inhibitor of SAM‐dependent methyltransferases, and SAH accumulation thereby impairs cell growth (Christopher et al., 2002). Previously, we showed that exogenous SAM improved the growth of the SAH hydrolase mutant *sah1*‐*1*, which accumulates high levels of SAH, suggesting that SAM is protective against SAH‐dependent growth inhibition (Mizunuma et al., 2004). Therefore, we speculate that SAH supplementation can increase SAM synthesis through an unknown mechanism. Since SAM synthesis requires Met (Figure 1a), stimulating SAM production can decrease the quantity of intracellular Met. Notably, among the amino acids, Met exhibited significantly reduced levels after SAH supplementation (Figure S1b).

To investigate whether the decrease in intracellular Met was due to accelerated consumption, we substituted L‐[methyl‐^13^C]Met in the culture medium for Met and followed its fate with or without addition of SAH. SAH supplementation significantly decreased [methyl‐^13^C]Met and increased [methyl‐^13^C]SAM intracellularly compared with the control (Figure 1d,e). Furthermore, extracellular metabolomic data showed that after SAH treatment [methyl‐^13^C]Met levels were comparable with that of the control (Figure 1f). These results suggest that SAH reduces Met levels by converting endogenous Met to SAM.

The lower Met content in SAH‐treated cells suggests that longevity from SAH supplementation can induce a MetR state. Hence, since MetR extends chronological lifespan (CLS) (Fabrizio & Longo, 2007) in an autophagy‐dependent manner (Plummer & Johnson, 2019; Ruckenstuhl et al., 2014), we investigated the effect of SAH on autophagy by monitoring the GFP‐Atg8 cleavage assay (Nair et al., 2011). SAH treatment increased degradation of the autophagy marker GFP‐Atg8 to yield free GFP (Figure 1g), suggesting that SAH administration promotes autophagy. Furthermore, since TORC1 negatively regulates autophagy (Shimobayashi & Hall, 2014), we tested whether SAH inhibits TORC1. In WT cells, treatment with SAH reduced the phosphorylation of Rps6, a homolog of ribosomal protein S6 (Figure 1h), which is phosphorylated by TORC1 (Wullschleger & Hall, 2006), suggesting that SAH reduces levels of TORC1 activity. Additionally, the CLS of *tor1*Δ and *atg7*Δ (deletion mutant of an essential autophagic machinery component) cells was not prolonged compared with WT cells treated with SAH (Figure 1i). Thus, consistent with the induction of MetR, SAH extends lifespan through the inhibition of TORC1 and activation of autophagy.

Subsequently, to determine whether SAH acts as an anti‐aging metabolite in a metazoan, we investigated its effects on the nematode *C*. *elegans*. SAH treatment extended the lifespan of WT animals in a concentration‐dependent manner, with 50 μM SAH inducing the most significant increase (Figure 2a). We also obtained similar results with 50 μM SAH in the absence of the reproduction blocker fluorodeoxyuridine (FUdR), or when SAH was supplemented only during adulthood. The latter finding ruled out a role for possible developmental effects (Figures S2a,b). Notably, SAH did not affect food consumption, brood size, or viability (Figure S2c,d,e). Additionally, the longevity‐extending effects of SAH were independent of bacterial metabolism (Figure S2f). SAH also partially prevented the aging‐associated decrease in physical capacity (Figure S2g). Altogether, these results suggest that SAH mediates phylogenetically conserved anti‐aging effects.

**FIGURE 2:**
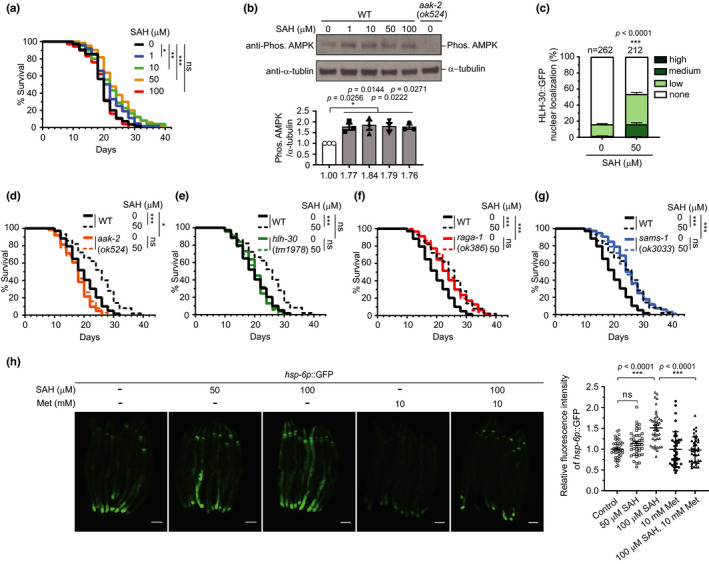
SAH extends lifespan via activation of AMPK, inhibition of mTORC1, and activation of autophagy in *C*. *elegans*. Representative survival curve (a) or relative AMPK phosphorylation level (b) of N2 WT animals in *C*. *elegans*, either untreated or treated with 1, 10, 50, 100 µM SAH. Mean ± SEM, *n* = 3, one‐way ANOVA with Tukey's correction. (c) Quantification of HLH‐30::GFP nuclear localization. *n* = number of worms. Mean ± SEM, chi‐square test. (d–g) Representative survival curves. (h) Representative images of *hsp*‐*6p*::GFP. Scale bar = 100 µm. Quantification of relative GFP intensity in the intestine is shown. Mean ± SEM, *n* = 40, one‐way ANOVA with Tukey's correction. (a–h) ns, not significant; *p < 0.05; **p < 0.01; ***p < 0.001. (a, d–g) Statistical analyses are shown in Table S3

Similar to findings in yeast (Ogawa et al., 2016), SAH supplementation increased the phosphorylation of AAK‐2, a *C*. *elegans* homolog of the catalytic AMPK subunit (Figure 2b). HLH‐30, an orthologue of the human transcription factor TFEB, is a master regulator that promotes autophagy (Settembre et al., 2011). Thus, we examined the autophagy activity by monitoring a GFP‐tagged HLH‐30 that translocates to the nucleus upon mechanistic TORC1 (mTORC1) inhibition (Settembre et al., 2012). SAH induced HLH‐30 nuclear accumulation (Figure 2c), suggesting that it can reduce mTORC1 activity and promote autophagy. Subsequently, to investigate how the SAH extends lifespan, we used the loss‐of‐function mutant strains *aak*‐*2(ok524)* (Apfeld et al., 2004), *hlh*‐*30(tm1978)* (Visvikis et al., 2014), and *raga*‐*1(ok386)* (Schreiber et al., 2010). No effect of SAH on lifespan extension in these mutants was observed, suggesting that SAH extends lifespan through a mechanism dependent on AMPK, mTORC1, and autophagy (Figure 2d,e,f).

*sams*‐*1* encodes an evolutionarily conserved SAM synthetase, knockdown of which extends lifespan (Hansen et al., 2005). SAH does not increase median lifespan further in *sams*‐*1(ok3033)*, a loss‐of‐function mutant allele of *sams*‐*1* (Walker et al., 2011) (Figure 2g), consistent with the idea that SAH extends lifespan through SAM synthesis. These results suggest that lifespan extension in the *sams*‐*1* strain, which is unable to produce SAM, is likely to occur through a mechanism entirely different from MetR.

Additionally, the expression of HSP‐6, an orthologue of the mitochondrial chaperone mitochondrial Hsp70, is induced by MetR through its induction of the mitochondrial unfolded protein response (UPR^mt^) (Amin et al., 2020). Supplementation with 100 μM SAH significantly increased the expression level of *hsp*‐*6p*::GFP (Figure 2h). Furthermore, this increase was suppressed upon Met supplementation, consistent with a model of MetR in *C*. *elegans*.

In conclusion, our results suggest that SAH extends lifespan by inducing MetR or mimicking its downstream effects. Since the lifespan‐extending effects of SAH are conserved in yeast and nematodes, and MetR extends the lifespan of many species, exposure to SAH is expected to have multiple benefits across evolutionary boundaries. Our findings offer the enticing possibility that in humans the benefits of a MetR diet can be achieved by promoting Met reduction with SAH. The use of endogenous metabolites, such as SAH, is considered safer than drugs and other substances, suggesting that it may be one of the most feasible ways to prevent age‐related diseases.

## EXPERIMENTAL PROCEDURES

Full detailed methods and experimental procedures are available in Appendix S1.

## CONFLICT OF INTEREST

None declared.

## AUTHOR CONTRIBUTIONS

M.M. conceived the study and designed the experiments. T.O. and M.M. carried out *C*. *elegans* experiments. T.O., K.M., and Y.K. performed yeast experiments. M.K. performed [methyl‐^13^C]Met analyses. T.S. performed metabolome analyses. T.O. and Y.O. discussed the results and contributed to the improvement of the manuscript. T.K.B. and M.M. wrote the manuscript. T.K.B. and M.M. supervised the work.

## Supporting information

Supplementary Material

Fig S1‐S3

Table S1

Table S2

Table S3

## Data Availability

The data that support the findings of this study are available from the corresponding author upon reasonable request.
