# Supplementary material for: S‐adenosyl‐L‐homocysteine extends lifespan through methionine restriction effects
Source: Aging Cell. 2022 Apr 7;21(5):e13604. doi: 10.1111/acel.13604 (PMC9124299; doi:10.1111/acel.13604)
Supplement: Supplementary file 1 — Supplementary Material [file ACEL-21-e13604-s003.docx]

**SUPPORTING INFORMATION**

**EXPERIMENTAL PROCEDURES**

**Yeast strains and media**

The following yeast strains used in this study were all derivatives of W303: W303-1A (wild-type (WT); *MAT***a** *trp1-1 leu2-3,112 ade2-1 ura3-1 his3-11,15 can1-100*), YMA32 (*MAT***a** *tor1*Δ*::kanMX4*), YYK1 (*MAT***a** *atg7*Δ*::kanMX4*), and YMA92 (*MAT***a** *cyt1*Δ*::kanMX4*). Media used is as previously described (Mizunuma et al., 2004; Ogawa et al., 2016).

**Yeast gene disruption and strain construction**

The *tor1*Δ, *atg7*Δ, and *cyt1*Δ strains were constructed by gene replacement. Genomic DNA was isolated from the *tor1*Δ*::kanMX4*, *atg7*Δ*::kanMX4*, and *cyt1*Δ*::kanMX4* strains on a BY4741 background (Invitrogen). The PCR-amplified fragments of *tor1::kanMX4*, *atg7::kanMX4*, and *cyt1::kanMX4* were used to transform the W303-1A.

**Chronological Lifespan (CLS) Assay for yeast**

Yeast CLS analysis was conducted on synthetic dextrose complete (SDC) liquid medium as previously described (Fabrizio et al., 2003; Ogawa et al., 2016). Briefly, SDC cultures grown overnight were diluted (2 x 10^6^ cells/ml) in fresh SDC medium and incubated at 28ºC with shaking at 180 rpm. Viability was measured using plating aging cells onto yeast extract (Bacto, 212750), peptone (Bacto, 211677), and dextrose (Nacalai Tesque, 16806-54) (YPD) plates and monitoring Colony Forming Units (CFUs) starting from day three, which was considered the initial survival (100%). All data were represented as the average of three independent experiments performed simultaneously. At least two sets of CLS experiments were conducted with similar outcomes. CLS assays were conducted using SDC medium [0.17% yeast nitrogen base w/o amino acids and ammonium sulfate (Difco, 233520); 0.5% ammonium sulfate (Sigma, 01-5110-5); 2% glucose (Nacalai Tesque, 16806-54); amino acids to a final concentration of 20 mg/l (adenine (Sigma, A9126), arginine (Kanto Chemical, 01434-30), histidine (Sigma, 13-1160-2), methionine (Nacalai Tesque, 21719-02), tryptophan (Kanto Chemical, 40339-30), and uracil (Sigma, U0750)), 30 mg/l (isoleucine (Sigma, 15-2120-2), leucine (Sigma, 18-1010-2), lysine (Sigma, 18-1900-2), and tyrosine (Sigma, 30-5530-2)), 60 mg/l (phenylalanine (Kanto Chemical, 32100-30)), and 150 mg/l (valine (Sigma, 33-0090-2))]. GraphPad Prism 9 (GraphPad Software) was used to compare CLS, and *p* values were obtained from a two-way ANOVA with time and strain used as independent factors.

**Growth conditions for metabolome analysis of yeast**

WT cells were cultured in SDC liquid medium. However, since SAH-treated cells had slower growth rate than the untreated cells (Figure S3), comparisons were made with cells cultured to the same OD_600_ values (∼0.4) rather than those cultured simultaneously. Cells were grown to log phase (A600 nm = 0.2) in SDC liquid medium at 25°C. SAH was added to SDC medium to 1 mM and cultured for another eight hours (A600 nm = 0.4). As a control, cells were grown log phase in SDC medium at 25°C (A600 nm = 0.4).

**Extraction of the intracellular metabolite from yeast**

Metabolite extraction was conducted using the previously described modification (Soga et al., 2003). Cells were harvested from the culture medium (OD600 = 30) and filtered through a 0.45 µm pore size filter. The internal cationic and anionic standards were methionine sulfone and 2-morpholinoethanesulfonic acid, respectively. The lyophilized samples were dissolved in 50 µl Milli-Q water and subjected to capillary electrophoresis-time-of-flight mass spectrometry (CE-TOFMS) analysis.

**Method for metabolome analysis**

*Instrumentation*: All capillary electrophoresis mass spectrometry (CE-MS) experiments were conducted using Agilent 7100 CE capillary electrophoresis (Agilent Technologies, Waldbronn, Germany), Agilent 6230 LC/MSD TOF system (Agilent Technologies, Palo Alto, CA, USA), an Agilent1100 series binary HPLC pump, and the G1603A Agilent CE-MS adapter- and G1607A Agilent CE-ESI-MS sprayer kit. For anionic metabolite analysis, the original Agilent stainless Electrospray ionization (ESI) needle was replaced with the Agilent G7100-60041 platinum ESI needle (Soga et al., 2009). Agilent MassHunter Workstation was used to conduct system control and data acquisition, and data analysis was done using the Keio MasterHands software.

*Cationic metabolite analysis by CE-MS*: Separations were conducted in a fused silica capillary (50 μm i.d. x 100 cm total length) filled with 1 M formic acid as the electrolyte (Soga et al., 2003; Soga et al., 2006). About 5 nl sample solution was injected at 50 mbar for five seconds, and 30 kV of voltage was applied. The capillary temperature was maintained at 20°C and the sample tray was cooled below 5°C. Methanol-water (50% v/v) containing 0.01 μM Hexakis (2,2-difluoroethoxy) phosphazene was delivered as the sheath liquid at 10 μl/min. ESI-time-of-flight mass spectrometry (ESI-TOFMS) was performed in the positive ion mode and the capillary voltage was set at 4,000 V. A flow rate of heated dry nitrogen gas (heater temperature 300°C) was maintained at 7 psig. In TOFMS, the fragmentor-, skimmer-, and Oct RFV voltage were set at 75 V, 50 V, and 500 V, respectively. Automatic recalibration of each acquired spectrum was conducted using reference masses of reference standards. The ^13^C isotopic ion of a protonated methanol dimer ([2MeOH + H]^+^, m/z 66.0631) and Hexakis(2,2-difluoroethoxy)phosphazene ([M + H]^+^, m/z 622.0290) provided the lock mass for exact mass measurements (Satoh et al., 2017).

*Anionic metabolite analysis by CE-MS*: A commercially available COSMO(+) (chemically coated with cationic polymer) capillary (50 μm i.d. x 105 cm total length) (Nacalai Tesque, Kyoto, Japan) was used with a 50 mM ammonium acetate solution (pH 8.5) as the electrolyte (Satoh et al., 2017; Soga et al., 2009). Sample solution (30 nl) was injected at 50 mbar for 30 sec and -30 kV of voltage was applied. Ammonium acetate (5 mM) in 50% methanol-water (v/v) containing 0.01 μM Hexakis(2,2-difluoroethoxy)phosphazene was delivered as the sheath liquid at 10 μl/min. ESI-TOFMS was conducted in the negative ion mode; the capillary voltage was set at 3,500 V. For TOFMS, the fragmentor-, skimmer-, and Oct RFV voltage were set at 100 V, 50 V, and 500 V, respectively. Automatic recalibration of each acquired spectrum was conducted using reference masses of reference standards, i.e., ^13^C isotopic ion of deprotonated acetic acid dimer ([2CH_3_COOH-H]^-^, m/z 120.0384), and Hexakis + deprotonated acetic acid (m/z 680.03554) provided the lock mass for exact mass measurements

*Statistical analysis:* MetaboAnalyst software ver. 5.0 was used for statistical analysis (Pang et al., 2021). Categories that have missing values were excluded. Data scaling was performed with mean-centered and divided by the standard deviation of each variable.

**Growth conditions with [methyl-^13^C]Met for metabolome analysis of yeast**

WT cells were preincubated in SD-Met liquid medium containing 20 mg/L [methyl-^13^C]Met (Cambridge Isotope Laboratories, CLM-206-1) at 25°C overnight. The cells were then grown in a fresh SD-Met liquid medium containing 20 mg/L [methyl-^13^C]Met liquid medium at 25°C until the logarithmic growth phase (A600 nm = 0.2). These procedures were designed to ensure that the only external source of Met was [methyl-^13^C]Met. The culture medium was then split into two, and 1 mM SAH solution was added to one of them, and the cells were cultured for another eight hours (A600 nm = 0.4). The other was used as a control and cultured until the logarithmic growth phase (A600 nm = 0.4). The [methyl-^13^C]Met and [methyl-^13^C]SAM content were expressed as nmol per mg dry cell weight (DCW).

**Extraction of intracellular [methyl-^13^C]Met and [methyl-^13^C]SAM from yeast**

Extraction of SAM and SAH was conducted as previously described (Christopher et al., 2002). The cells were harvested (total OD600 nm = 15), washed twice with 20 ml cold water, and then extracted with 1 ml 10% perchloric acid (Fujifilm Wako Chemical, 160-05755) for one hour at room temperature. The supernatant was diluted with MilliQ-grade water, and the samples were filtered for CE-TOFMS.

**GFP-ATG8 processing assay**

Cells harboring the GFP-Atg8 expressing plasmid were grown to log phase (A600 nm = 0.2) in liquid SD medium lacking uracil at 25ºC. SAH solution was added to SDC medium to 1 mM, and the cells were incubated for another eight hours (A600 nm = 0.4). For the control, cells were grown to log phase (A600 nm = 0.4) in liquid SDC medium at 25ºC. Cells growth was stopped by adding TCA (Sigma, 30-3890-5) to about 9% final concentration and putting on ice for at least five minutes before cells were pelleted. The cells were obtained by centrifugation, washed twice with cold acetone (Nacalai Tesque, 00310-95), and dried in a speed-vac. Pellets were resuspended in 100 µl of urea buffer (50 mM Tris (Sigma, T1378) [pH 7.5], 1 mM EDTA (Sigma, 09-1420-5), 6 M urea (Kanto Chemical, 43009-01), 1% SDS (Nacalai Tesque, 08933-05), 1 mM PMSF (Sigma, P7626), 1x complete protease inhibitor cocktail (Roche, 11873580001), and 0.1% Tween 20 (Bio-Rad, 1706531)) and lysed by vortexing with an equal volume of glass beads at room temperature, with subsequent heating for ten minutes at 65ºC. Further analysis was conducted using SDS-PAGE and immunoblotting using anti-GFP antibody (Roche, 11814460001, 1:1000) and anti-PSTAIR antibody (Novus bio., NB120-10345, 1:1000). ImageJ was used to quantify signals for Western blotting results, and GraphPad Prism 9 was used for statistical analysis.

**Detection of Rps6 activation**

WT cells were grown to log phase (A600 nm = 0.2) in the SDC liquid medium at 25°C. SAH was added to SDC medium to 1 mM and cultured for another eight hours (A600 nm = 0.4). As a control, cells were grown log phase in SDC medium at 25°C (A600 nm = 0.4). Cells were then harvested, washed with 1ml TEG +PPi buffer (50 mM Tris (Sigma, T1378)-HCl (Nacalai Tesque, 18321-05), 1 mM EDTA (Sigma, 09-1420-5) [pH 7.5], 10% glycerol (Nacalai Tesque, 17018-25), 30 mM NaCl (Nacalai Tesque, 31320-76), 10 mM NaF (Fujifilm Wako Chemical, 192-01972), 1 mM sodium orthovanadate (Na3VO4) (Fujifilm Wako Chemical, 198-09752), 1 mM PMSF (Sigma, P7626), 1 mM DTT (Nacalai Tesque, 14112-81), 1x complete protease inhibitor cocktail (Roche, 11873580001), and 0.1% Tween 20 (Bio-Rad, 1706531)). The precipitate was frozen in liquid nitrogen and stored at -80°C. The frozen precipitate was dissolved on ice and gently resuspended in SDS-PAGE sample buffer, and boiled for ten minutes. The eluted proteins were resolved by SDS-PAGE and detected by anti-Phospho-S6 Ribosomal Protein (Ser235/236) antibody (Cell signaling Tech., 4858, 1:1000) and anti- PSTAIR antibody (Novus bio., NB120-10345, 1:1000). ImageJ was used to quantify signals for Western blotting results, and GraphPad Prism 9 was used for statistical analysis.

***Caenorhabditis elegans* strains and maintenance**

*C. elegans* strains were grown on solid nematode growth media (NGM) plates containing 10 mg/L streptomycin seeded with OP50 *Escherichia Coli* bacteria at 20ºC. The following strains were used in this study, N2: WT Bristol isolate, RB754: *aak-2(ok524) X*, JIN1375: *hlh-30(tm1978*) *IV*, VC222: *raga-1(ok386) II*, RB2240: *sams-1(ok3033) X*, MAH235: *sqls19 [hlh-30p::hlh-30::GFP + rol-6(su1006)],* SJ4100: *zcls13 [hsp-6p::GFP* *+ lin-15(+)]*. Synchronous populations were obtained using hypochlorite treatment, unless otherwise noted.

**SAH and Met medium preparation**

Here, 1 mM SAH (Sigma, A9384) dissolved in sterilized water was prepared. Autoclaved NGM was cooled down to be around 45ºC and mixed with 10 mg/L Streptomycin and SAH to be concentration was indicated in each experiment, unless otherwise indicated. Control plates were mixed with sterilized water with the same amount of SAH solution. OP50 were incubated for 12-16 hours in Luria-Bertani (LB) (Sigma, L7658) with 10 mg/L Streptomycin at 37ºC. The bacterial culture was mixed with 1 mM SAH to be a concentration indicated in each experiment and seeded on NGM plates. Plates were placed at room temperature through overnight and stored at 4ºC until it was needed. If necessary, bacteria-seeded plates were dried in a laminar flow hood. 100 mM Met (Sigma, M8439) dissolved in sterilized water was prepared. Autoclaved NGM was cooled down to be around 45ºC and mixed with 10 mg/L Streptomycin and Met to be concentration was indicated in each experiment. Control plates were mixed with sterilized water with the same amount of Met solution.

**Lifespan analysis of *C. elegans***

All lifespan assays were conducted at 20 ºC on solid NGM plates without antibiotics. OP50 was incubated in LB with 10 mg/L for 12-16 hours at 37ºC and seeded on NGM plates. All worms were passaged for at least two generations before measurement. Day one of adulthood strains were synchronized by six hours of timed egg-lay on NGM, either untreated or treated with SAH. After three days, 20-30 synchronized young adults were transferred to NGM plates containing 60 µM 5-fluoro-2’deoxyuridine (FUdR), either untreated or treated with SAH unless otherwise indicated. After 4-5 days, worms were transferred to flesh NGM plates not to be starved. Survival was plotted with the young adult as time-point = 0 and was scored every day or every other day. Worms were censored when they were crawled off the plate, hatched inside, or lost vulva integrity. Survival curves were generated using the GraphPad Prism 7. *p* values were determined by log-rank (Mantel-Cox) test. At least three independent replicates for the main figures or two independent replicates for supplemental figures were examined for each data set. All lifespan data are available in Table S3.

For the assay without FUdR, 20-30 synchronized young adults were transferred to NGM plates, either untreated or treated with SAH, and were transferred onto fresh plates every day until they stopped laying eggs.

For the assay with UV-killed bacteria, incubated bacteria with LB containing 1 mg/L streptomycin at 37ºC were spun down, and resuspended with LB to be OD = 10. Bacteria were seeded 200 µl on NGM plates, and plates were placed in the hood until they dried. Dried plates were exposed to UV for 30 min twice with a maximal energy of a Stratagene UV Stratalinker 2400 (La Jolla, CA, USA). UV-killing was verified by failure to form colonies when streaked onto LB plates.

**AMPK phosphorylation assay**

Three thousand synchronized eggs were incubated for two days. Worms at the L4 stage were collected and washed twice using M9 buffer. Worms were resuspended with 100 µl of protein lysis buffer (150 mM NaCl (Sigma, S3014), 1% NP-40 (Sigma, 74385), 50 mM Tris-HCl (pH 8.0) (Sigma, T6066), containing PhosSTOP (Roche, 4906845001) and cOmplete, Mini Protease Inhibitor Cocktail (Roche, 11836153001). Worms were lysed using sonication for ten seconds twice. The worm lysate was spun down with maximal speed at 4ºC, and the supernatant was stored at -20ºC. Protein concentrations were measured using standard BCA assay with Pierce BCA Protein Assay Kit (Thermo Fisher, 23227) and all samples were adjusted to be equalized. LDS sample buffer (Invitrogen, NP0007) was added and boiled at 95ºC for five minutes, and 16 µg of samples were loaded and separated using SDS-PAGE, and transferred to PVDF membranes. Blots were blocked for one hour with Starting Block T20 (TBS) Blocking Buffer (Thermo Fisher, 37543) and probed with anti-phosphorylated AMPK (Cell Signaling Technology, 2535, 1:1000) or anti-α tubulin (Sigma, T9026, 1:5000) primary antibodies overnight at 4ºC. HRP conjugated anti-rabbit (Thermo Fisher, A27036) and anti-mouse (Thermo Fisher, A28177) secondary antibodies were used for 1 hour at room temperature. Protein signals were captured using a BioRad ChemiDoc imaging system.

**HLH-30::GFP localization assay**

HLH-30::GFP localization was performed by using MAH235. Synchronized eggs were placed on NGM plates with OP50 bacteria, either untreated or treated with SAH. After three days, day one of adulthood was mounted on objective slides with 2% agar pads using 20 mM Tetramisole (Sigma, T1512) for immobilization. Fluorescence images were obtained using an Axio Imager M2 microscope and 10X/0.25 objective (Zeiss) and processed using ZEN 2012 software. All samples were taken within five minutes of mounting. Acquisition parameters were kept identical across all samples. Images were scored as follows: none, GFP diffused into the cytoplasm and no intensity difference between the cytoplasm and nuclei; low, GFP localized in nuclei stronger than the cytoplasm in some intestinal cells; medium, GFP localized in nuclei stronger than cytoplasm in all intestinal cells; high, GFP localized only in nuclei in all intestinal cells.

***hsp-6p*::GFP reporter assay**

*hsp-6p*::GFP reporter assay was performed by using SJ4100 which is fused HSP-6 promoter region with GFP. Synchronized eggs by 6 hours of timed egg-lay were placed on NGM plates with OP50 bacteria, either untreated or treated with SAH with or without Met. After 3 days, day one of adulthood was mounted on objective slides with 2% agar pads using 20 mM Tetramisole for immobilization. Fluorescence images were obtained using an Axio Imager M2 microscope and 10X/0.25 objective and processed using ZEN 2012 software. Acquisition parameters were kept identical across all samples. Images were scored by measuring GFP intensity in intestine with ImageJ.

**Brood size assay**

Synchronized eggs by 6 hours of timed egg-lay were placed on NGM plates with OP50 bacteria, either untreated or treated with SAH. After 2 days, L4 worms were transferred to new small NGM plates (35 mm x 10 mm) each, with OP50 bacteria, either untreated or treated with SAH. All worms were transferred to new small plates every day until they stop laying eggs. After 24 hours of transferring worms, dead eggs and live progeny were counted.

**Food consumption assay**

Food consumption assay was conducted as previously described (Wu et al., 2019). Briefly, the OP50 bacteria that grew overnight at 37ºC were obtained, and resuspended with S-basal completely buffer containing 5 mg/L cholesterol, 50 mg/L ampicillin, 10 mg/L kanamycin, 1 mg/L tetracycline, and 50 mg/L nystatin to reach OD_600_ = 3.0. OP50 was kept at 4ºC for 5-14 days and taken out four hours before use. Synchronized eggs of six hours of timed egg-lay were cultured for two days on regular NGM plates with or without SAH at 20ºC. Animals were washed twice using S-basal buffer and transferred to a 24-well plate containing 900 μl prepared antibiotic-arrested OP50, in addition to 100 μl 500 µM SAH or sterilized water, with 30-50 worms, which was resuspended in 30 μl S-basal and placed in each well. After 5-7 days incubation at 20ºC, OD_600_ of bacteria concentration were measured. For each experiment, five replicates, at least 150 worms in total have been evaluated. The relative food intake was determined by the change in OD_600_ for each well, normalized to the number of worms.

**Body bends assay**

Body bends assay was conducted using the modified method from described protocol (Bansal et al., 2015; Hahm et al., 2015). Synchronized eggs were incubated on NGM plates, either untreated or treated with SAH for two days. Worms at the L4 stage were transferred to new NGM plates containing 60 µM FuDR, either untreated or treated with SAH and were incubated at 20ºC. On day one, five, and ten of adulthood, worms were transferred to 96-well plates filled with M9 buffer (a worm per well). Swimming of worms was recorded for 30 sec immediately after transferring using a stereomicroscope (Optika SMZ-4) with Olympus DP72 camera and cellSens Standard software (Olympus). The recorded image were analyzed using ImageJ plugin Worm-tracker (wrMTrck) (Nussbaum-Krammer et al., 2015). At least 15 worms were analyzed in each experiment.

**Data collection and statistical analysis.** No statistical methods were used to predetermine the sample size. Fluorescence reporter assays were blindly scored. All other experiments were not scored blindly. Metabolome experiments (Figures 1b-1f) were conducted in triplicate in independent experiments and data were analyzed using the mean of the triplicate samples. All other experiments were repeated at least twice with similar results each time. The data represent biological replicates. Appropriate statistical tests were used for every figure. Data are represented as mean ± S.E.M. or ± S.D.; *p* value less than 0.05 were defined as statistically significant.

**References S1 Additional citations**

Bansal, A., Zhu, L. J., Yen, K., & Tissenbaum, H. A. (2015). Uncoupling lifespan and healthspan in Caenorhabditis elegans longevity mutants. *Proceedings of the National Academy of Sciences of the United States of America*, *112*, E277-E286. <https://doi.org/10.1073/pnas.1412192112>

Fabrizio, P., Liou, L.-L., Moy, V. N., Diaspro, A., Valentine, J. S., Gralla, E. B., & Longo, V. D. (2003). SOD2 functions downstream of Sch9 to extend longevity in yeast. *Genetics, 163*, 35-46. <https://doi.org/10.1093/genetics/163.1.35>

Hahm, J. H., Kim, S., DiLoreto, R., Shi, C., Lee, S. J., Murphy, C. T., & Nam, H. G. (2015). C. elegans maximum velocity correlates with healthspan and is maintained in worms with an insulin receptor mutation. *Nature Communications, 6*, 8919. <https://doi.org/10.1038/ncomms9919>

Nussbaum-Krammer, C. I., Neto, M. F., Brielmann, R. M., Pedersen, J. S., & Morimoto, R. I. (2015). Investigating the spreading and toxicity of prion-like proteins using the metazoan model organism C. elegans. *Journal of Visualized Experiments, 95*, 52321. <https://dx.doi.org/10.3791/52321>

Pang, Z., Chong, J., Zhou, G., de Lima Morais, D. A., Chang, L., Barrette, M., Gauthier, C., Jacques, P., Li, S., & Xia, J. (2021). MetaboAnalyst 5.0: narrowing the gap between raw spectra and functional insights. *Nucleic Acids Research, 49*, W388–W396 <https://doi.org/10.1093/nar/gkab382>

Satoh, K., Yachida, S., Sugimoto, M., Oshima, M., Nakagawa, T., Akamoto, S., Tabata, S., Saitoh, K., Kato, K., Sato, S., Igarashi, K., Aizawa, Y., Kajino-Sakamoto, R., Kojima, Y., Fujishita, T., Enomoto, A., Hirayama, A., Ishikawa, T., Taketo, M. M., Kushida, Y., Haba, R., Okano, K., Tomita, M., Suzuki, Y., Fukuda, S., Aoki, M., & Soga, T. (2017). Global metabolic reprogramming of colorectal cancer occurs at adenoma stage and is induced by MYC. *Proceedings of the National Academy of Sciences of the United States of America*, *114*, E7697-E7706. <https://doi.org/10.1073/pnas.1710366114>

Soga, T., Baran, R., Suematsu, M., Ueno, Y., Ikeda, S., Sakurakawa, T., Kakazu, Y., Ishikawa, T., Robert, M., Nishioka. T., & Tomita, M. (2006). Differential metabolomics reveals ophthalmic acid as an oxidative stress biomarker indicating hepatic glutathione consumption. *Journal of Biological Chemistry, 281*, 16768-16776. <https://doi.org/10.1074/jbc.M601876200>

Soga, T., Igarashi, K., Ito, C., Mizobuchi, K., Zimmermann, H. P., & Tomita, M. (2009). Metabolomic profiling of anionic metabolites by capillary electrophoresis mass spectrometry. *Analytical Chemistry, 81*, 6165-6174. <https://doi.org/10.1021/ac900675k>

Soga, T., Ohashi, Y., Ueno, Y., Naraoka, H., Tomita, M., & Nishioka, T. (2003). Quantitative metabolome analysis using capillary electrophoresis mass spectrometry. *Journal of Proteome Research, 2,* 488-494. <https://doi.org/10.1021/pr034020m>

Wu, Z., Isik, M., Moroz, N., Steinbaugh, M. J., Zhang, P., & Blackwell, T. K. (2019). Dietary Restriction Extends Lifespan through Metabolic Regulation of Innate Immunity. *Cell Metabolism, 29*, 1192-1205.e8. <https://doi.org/10.1016/j.cmet.2019.02.013>

**Supplementary Figure Legends**

**Figure S1 Intracellular metabolic changes when SAH is added to WT cells shown in Figures 1b and 1c.** (a) Metabolite changes in the presence or absence of SAH in WT cells. Red and blue show increased and decreased metabolites, respectively. For a complete list, see Table S1. Mean ± S.D. (n = 3). **p* < 0.05; ***p* < 0.01; ****p* < 0.001 (two-sided unpaired *t*-test assuming equal variance). (b) SAH predominantly reduces only intracellular Met. Values of amino acids in WT cells were set to one, respectively, after which the amino acids values when SAH was added were compared. Mean ± S.D. (n = 3). **p* < 0.05; ***p* < 0.01; ****p* < 0.001 (two-sided unpaired *t*-test assuming equal variance).

**Figure S2 SAH supplementation extends lifespan by activating AMPK and inducing autophagy in *C. elegans* shown in Figure 2.** (a) The representative survival curve of WT animals, either untreated or treated with 50 µM SAH without FUdR. (b) Representative survival curve of WT animals, either untreated or treated with 50 µM SAH from the young adult stage. (c) Relative food consumption from the L4 stage until day 3-5 of adulthood, either untreated or treated with 50 µM SAH. Mean ± S.E.M. of 4-5 replicates in a representative experiment. ns, not significant (two-sided unpaired *t*-test with Welch’s correction). The number of brood sizes (d) or live progenies (e) of WT animals, either untreated or treated with 50 µM SAH. Mean ± S.E.M. Data are indicated due to the total number of tested worms with two independent experiments. ns, not significant (one-way ANOVA with Tukey’s correction). (f) Representative survival curve of WT animals, either untreated or treated with 50 µM SAH and UV-killed bacteria. (g) Body bends per second of WT animals, untreated or treated with 50 µM SAH. Mean ± S.E.M. Data are shown due to the total number of tested worms with two independent experiments. ***p* < 0.01 (two-way ANOVA with Tukey’s correction). (a, b, f) The representative set of data from two biological repeats is shown. *p* values were determined using the log-rank test. ns, not significant; **p* < 0.05; ***p* < 0.01; ****p* < 0.001. For each data sets, the number of individuals, repetition and statistical analysis is shown in Table S3.

**Figure S3 The effect of SAH supplementation on the growth of WT cells.** Growth rate and cell density of WT cells with (closed circle) or without (opened circle) SAH treatment is shown. WT cells cultured in an SDC medium to log phase (A600 nm = 0.2) were further cultured with or without 1 mM SAH. A representative set of data from two biological repeats is shown. Mean ± S.D. (n = 3).

**Supplementary Tables**

**Table S1 Metabolomics raw data shown in Figures 1b and 1c; Figure S1.**

**Table S2 Lifespan Analyses of *S. cerevisiae* shown in Figures 1i**

**Table S3 Lifespan Analyses of *C. elegans* shown in Figures 2a and 2d-g; Figures S2a, S2b, and S2f.**
