# Supplementary figures and images for: S‐adenosyl‐L‐homocysteine extends lifespan through methionine restriction effects
Source: Aging Cell. 2022 Apr 7;21(5):e13604. doi: 10.1111/acel.13604 (PMC9124299; doi:10.1111/acel.13604)

Figure S1

(a)

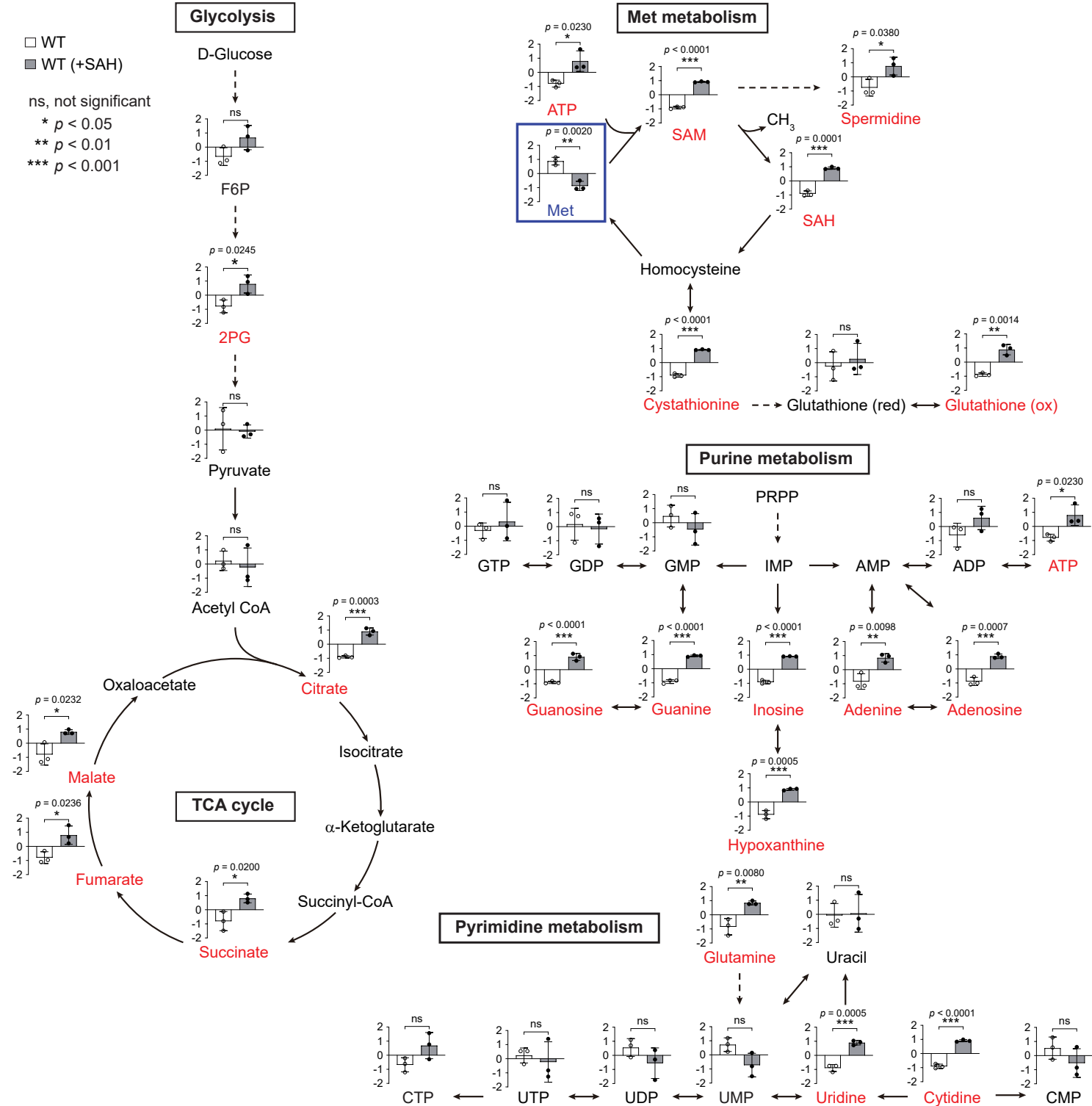

(b)

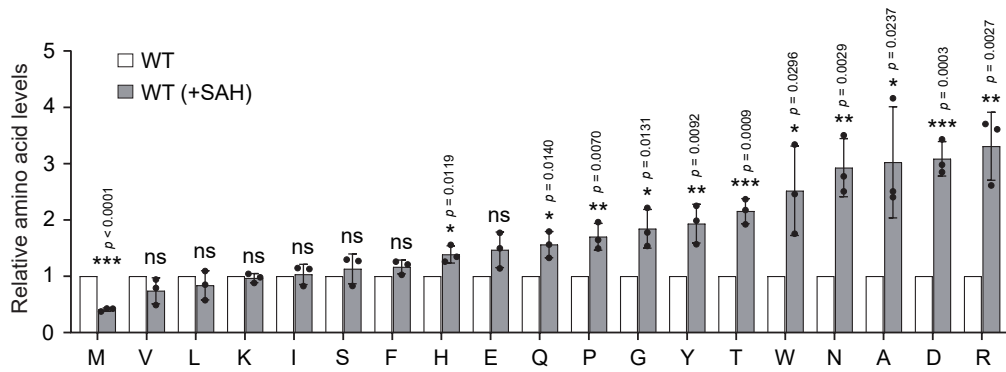

Figure S2

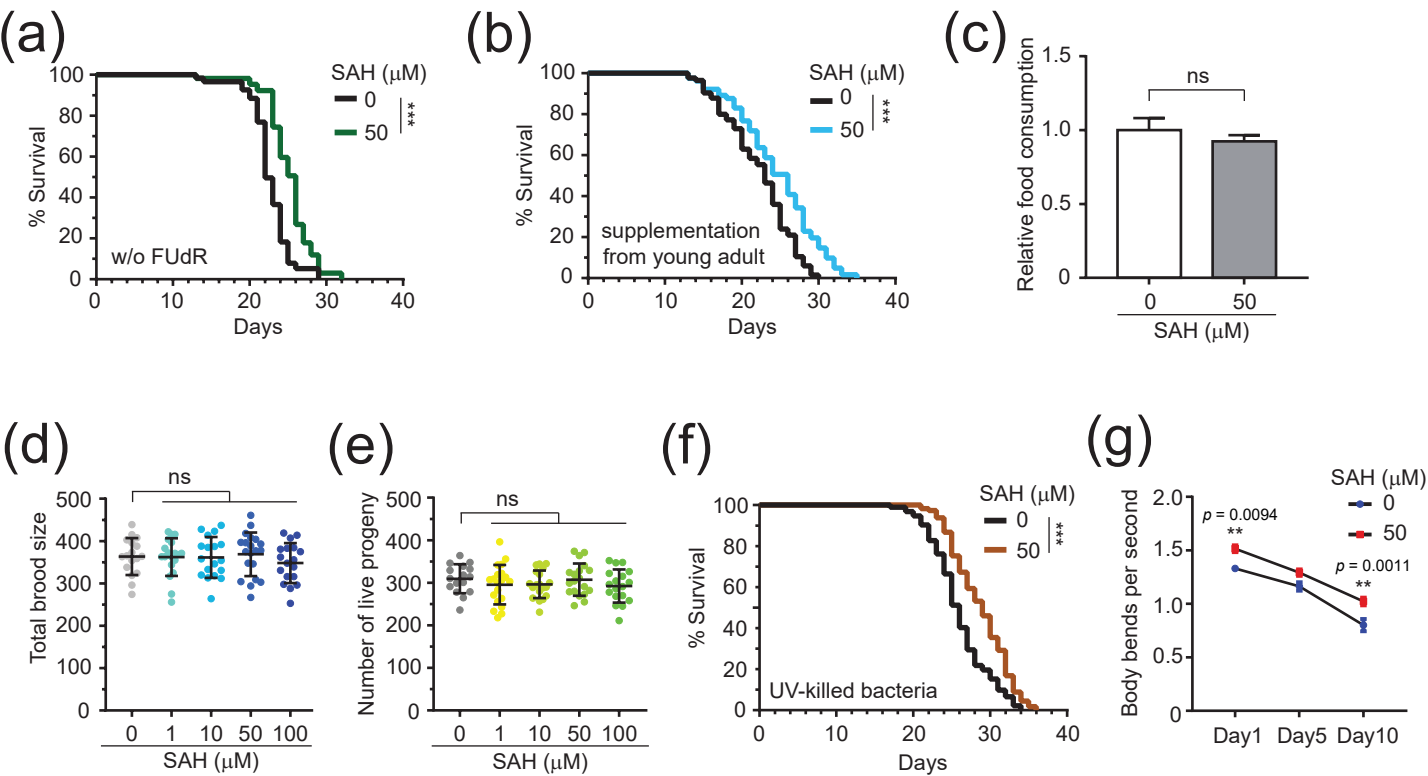

Figure S3

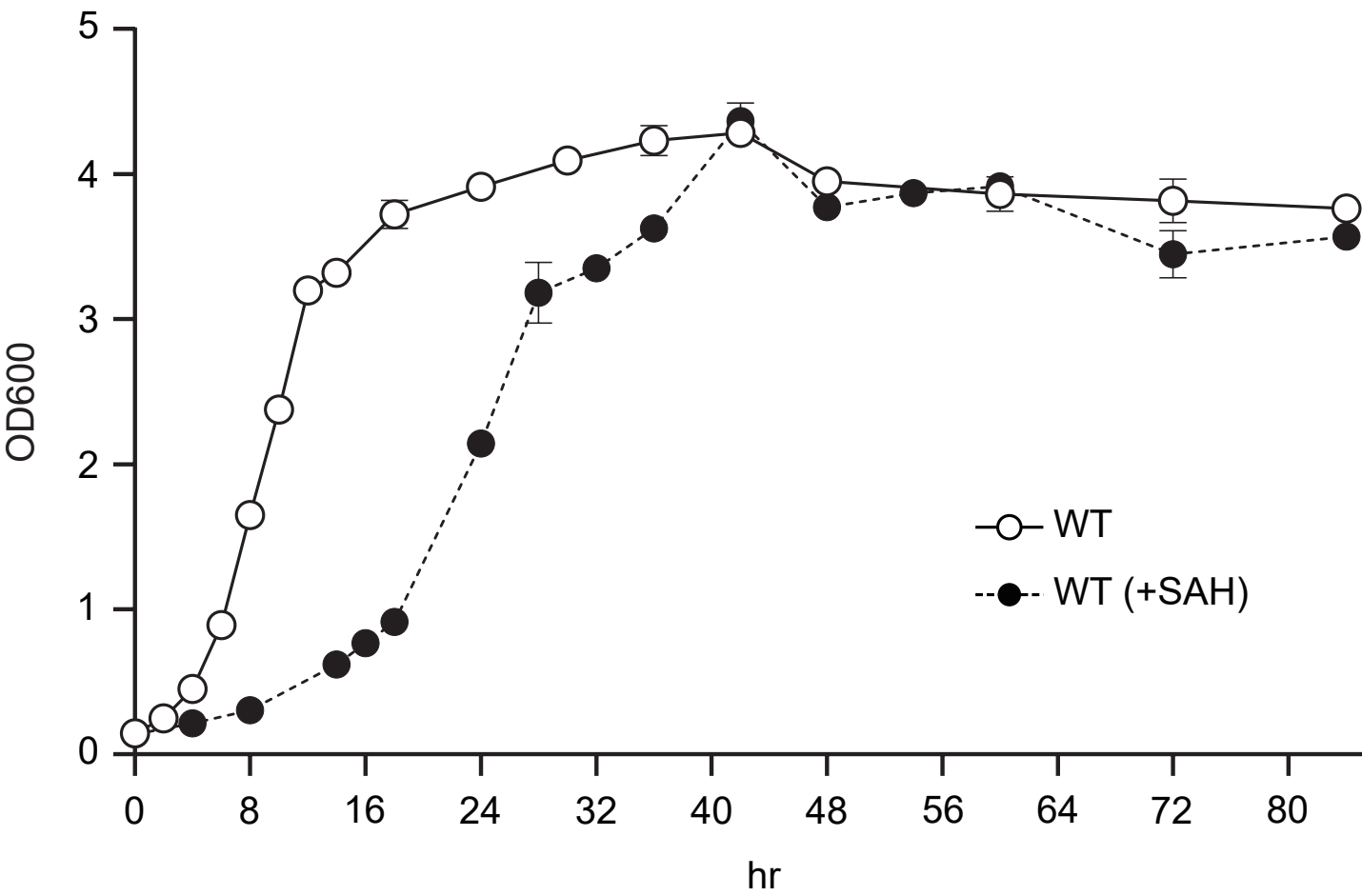

Supplement: Supplementary file 2 — Fig S1‐S3 [file ACEL-21-e13604-s005.pdf]
